# Building and Applying Quantitative Adverse Outcome Pathway Models for Chemical Hazard and Risk Assessment

**DOI:** 10.1002/etc.4505

**Published:** 2019-08-08

**Authors:** Edward J. Perkins, Roman Ashauer, Lyle Burgoon, Rory Conolly, Brigitte Landesmann, Cameron Mackay, Cheryl A. Murphy, Nathan Pollesch, James R. Wheeler, Anze Zupanic, Stefan Scholz

**Affiliations:** ^1^ US Army Engineer Research and Development Center Vicksburg Mississippi USA; ^2^ Environment Department University of York, Heslington York UK; ^3^ Toxicodynamics York UK; ^4^ Integrated Systems Toxicology Division, National Health and Environmental Effects Research Laboratory, Office of Research and Development US Environmental Protection Agency, Research Triangle Park North Carolina USA; ^5^ Joint Research Centre, Ispra Italy; ^6^ Unilever Safety and Environmental Assurance Centre, Sharnbrook Bedford UK; ^7^ Department of Fisheries and Wildlife Michigan State University East Lansing Michigan USA; ^8^ Mid‐Continent Ecology Division, National Health and Environmental Effects Laboratory, Office of Research and Development US Environmental Protection Agency Duluth Minnesota USA; ^9^ Dow AgroSciences Abingdon Oxfordshire UK; ^10^ Department of Environmental Toxicology Swiss Federal Institute for Aquatic Science and Technology Dübendorf Switzerland; ^11^ Department of Bioanalytical Ecotoxicology Helmholtz Centre for Environmental Research‐UFZ Leipzig Germany

**Keywords:** Quantitative adverse outcome pathways, Toxicokinetic/toxicodynamic modeling, Alternatives to animal testing, Predictive toxicology, Species extrapolation, Prioritization of chemicals

## Abstract

An important goal in toxicology is the development of new ways to increase the speed, accuracy, and applicability of chemical hazard and risk assessment approaches. A promising route is the integration of in vitro assays with biological pathway information. We examined how the adverse outcome pathway (AOP) framework can be used to develop pathway‐based quantitative models useful for regulatory chemical safety assessment. By using AOPs as initial conceptual models and the AOP knowledge base as a source of data on key event relationships, different methods can be applied to develop computational quantitative AOP models (qAOPs) relevant for decision making. A qAOP model may not necessarily have the same structure as the AOP it is based on. Useful AOP modeling methods range from statistical, Bayesian networks, regression, and ordinary differential equations to individual‐based models and should be chosen according to the questions being asked and the data available. We discuss the need for toxicokinetic models to provide linkages between exposure and qAOPs, to extrapolate from in vitro to in vivo, and to extrapolate across species. Finally, we identify best practices for modeling and model building and the necessity for transparent and comprehensive documentation to gain confidence in the use of qAOP models and ultimately their use in regulatory applications. *Environ Toxicol Chem* 2019;38:1850–1865. © 2019 The Authors. *Environmental Toxicology and Chemistry* published by Wiley Periodicals, Inc. on behalf of SETAC.

## INTRODUCTION

Traditionally, the hazard and risk assessment of chemicals has relied heavily on animal testing. In addition to a limited predictive capacity for human and environmental health effects, these tests can be time consuming, costly, and raise ethical concerns. Furthermore, it is not feasible to determine the health hazards of the thousands of different chemicals in commerce that lack toxicological data using animal tests (Dix et al. 2007). As a result, there is an increasing need for more cost‐effective, species‐specific, and mechanistic testing approaches, such as human in vitro cellular assays and other emerging technologies (Dix et al. 2007; National Research Council of the National Academies 2007; Cote et al. 2016). Because many of these technologies measure effects at the suborganismal level, new quantitative modeling approaches are needed to extrapolate measured toxicological effects to the whole organism or from test species (e.g., zebrafish) to species of concern (e.g., human).

**Figure 1:**
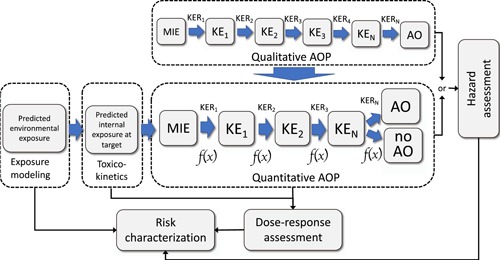
Use of quantitative adverse outcome pathways (AOPs) models in hazard and risk assessment. Quantitative AOPs (qAOPs) are developed from qualitative AOPs but have quantitative descriptors (*f*(*x*)) for key event (KE) relationships (KERs). Both AOPs and qAOPs can be used in hazard identification and assessment, but qAOP models are needed for dose–response assessments. Risk assessment applications combine qAOPs with chemical‐specific information and/or models that characterize the external and the corresponding internal concentration of chemical that is available to activate the molecular initiating event (MIE). (Note: *f*(*x*) may represent a mathematical or statistical function. AO = adverse outcome.)

Frameworks that support the plausibility and causal understanding of how chemical exposures lead to toxicity/adverse outcomes include the mode of action framework (Sonich‐Mullin et al. 2001; Meek et al. 2003; US Environmental Protection Agency 2005; Boobis et al. 2009; Meek et al. 2013) and, more recently, the adverse outcome pathway (AOP) concept (Ankley et al. 2010). The AOP framework has emerged as one potential way of integrating evidence from in vitro assays in the context of a pathway that leads to an adverse outcome. A key feature of the AOP framework is that it is chemically agnostic (not specific for one particular chemical), enabling one AOP to be used to describe the potential actions of a group of chemicals. An AOP describes a biological pathway that can be perturbed by a chemical or other stressor and captures the consecutive changes occurring at multiple biological levels that cause adverse effects of regulatory interest. In the AOP framework (Figure 1), the initiation of a molecular initiating event starts a cascade of key events causally linked by key event relationships (KERs) that lead to an adverse outcome (Ankley et al. 2010). A key event reflects a measurable change in a biological state that is necessary for the progression toward an adverse event. The KERs represent the regulatory, mechanistic, structural, and/or functional relationship between 2 key events and are supported by empirical data that provide information on dose response and temporality (Meek et al. 2014; Becker et al. 2017).

Textbox 1 **AOPs in the context of hazard and risk assessment**

Chemical risk assessment is a process that typically combines four different parts: hazard assessment, dose‐response assessment, exposure assessment and risk characterization. Hazard assessment identifies whether or not a chemical can cause an adverse effect. Dose‐response assessment characterizes the amount of a chemical needed to elicit an adverse effect often by identifying chemical concentrations or doses at which treated animals or assays diverge from controls (points of departure or POD). Exposure assessment estimates how and how much of a chemical is available to cause adverse effects in individuals or populations. Risk characterization integrates information on exposure, hazard, and dose‐response to estimate the likelihood of adverse effects in exposed individuals and/or populations. Adverse outcome pathways (AOPs) can be used in each of the 4 assessments. Qualitative AOPs systematically structure knowledge of the cascade of key events, from the interaction of a chemical with a receptor, enzyme, or other biological molecules (molecular initiating events) to an adverse outcome, thereby enabling their use in hazard assessment. Quantitative AOPs, when sufficient quantitative information is available to describe dose–response and/or response–response relationships among the molecular initiating event, the key event, and the adverse outcome, can be used to identify a point of departure for calculation of the external doses needed to cause a hazardous effect or adverse outcome in a dose–response assessment. As a result, both qualitative and quantitative AOPs can be useful in risk characterization by identifying, structuring, and integrating the available evidence for chemical hazards. However, only quantitative AOPs are useful for integrating dose–response assessment with exposure assessment by linking exposure to the amount of chemical needed to cause a point of departure in an AOP.

However, there remains a critical need to extend the AOP framework to support prediction of chemical doses or concentrations that would lead to adverse outcomes at the individual and population level (Kramer et al. 2011; Wheeler and Weltje 2015). Such an extension requires a detailed quantitative description of the relationships among the molecular initiating events, the key events, and the adverse effect (Conolly et al. 2017). This would enable the development of biomarkers that can lead to earlier diagnosis of disease and/or prediction of adverse effects that could be measured by in vitro assays (Perkins et al. 2015). The KERs are likely to already contain some degree of quantitative information that could be used to develop statistical relationships or mathematical functions to infer the state of the downstream key event from the known, measured, or predicted state of the upstream key event (Organisation for Economic Co‐operation and Development 2016). Moreover, the application of AOPs in dose–response assessment and risk characterization (see Textbox 1) will require linkage to chemical‐specific information such as toxicokinetics that describe how much chemical is available at the tissue or cellular level to affect a molecular initiating event (Scholz 2015). Some aspects of quantitative (q)AOPs, particularly with respect to hypothesis testing, have been recently described by Perkins et al. (2019). In the present review we focus on how the principles of quantitative models for AOPs can be established, and their potential advantages and applications.

### Development of quantitative AOP models

The relationships between key events in an AOP provide a description of how one event causes a change in, or transitions to, a second event. Quantitatively, a KER may be defined in terms of regressions between key events, response–response relationships, or dose‐dependent transitions. They may take the form of simple mathematical equations or sophisticated biologically based computational models that consider other modulating factors, such as compensatory responses, or interactions with other biological or environmental variables. Depending on the level and nature of the empirical data available, there is a continuum of AOPs from purely descriptive qualitative AOPs to qAOP models with detailed response–response relationships that allow one to infer the magnitude or probability of an adverse outcome. In the present review we define a full qAOP model as any mathematical construct that models the dose–response or response–response relationships of all KERs described in an AOP, a partial qAOP as a construct that models the dose/response–response relationships of more than one KER, and a quantitative KER as a construct that models a single dose/response–response relationship (Figure 1). The qAOP models support explicit incorporation of complex relationships, such as feedback loops, thresholds, and signaling cascades that are generally embedded in the key event or KER of descriptive AOPs. Models incorporating complex biological relationships can create predictions with greater biological fidelity to support hazard and risk assessment than models with simplified assumptions (Conolly et al. 2017).

The objective of our review is to describe how qAOP models can be developed and to provide examples of how they could be used in a hazard or risk assessment context. We describe how qAOP models can be built from qualitative AOPs, and how different modeling approaches can be applied to developing qAOPs, and we discuss the documentation requirements that can facilitate the use, communication, and acceptance of qAOP models. Finally, we discuss how these approaches can support regulatory decision making in hazard and risk assessment along with example qAOP applications.

## HOW TO BUILD A QUANTITATIVE MODEL FROM AN AOP

**Figure 2:**
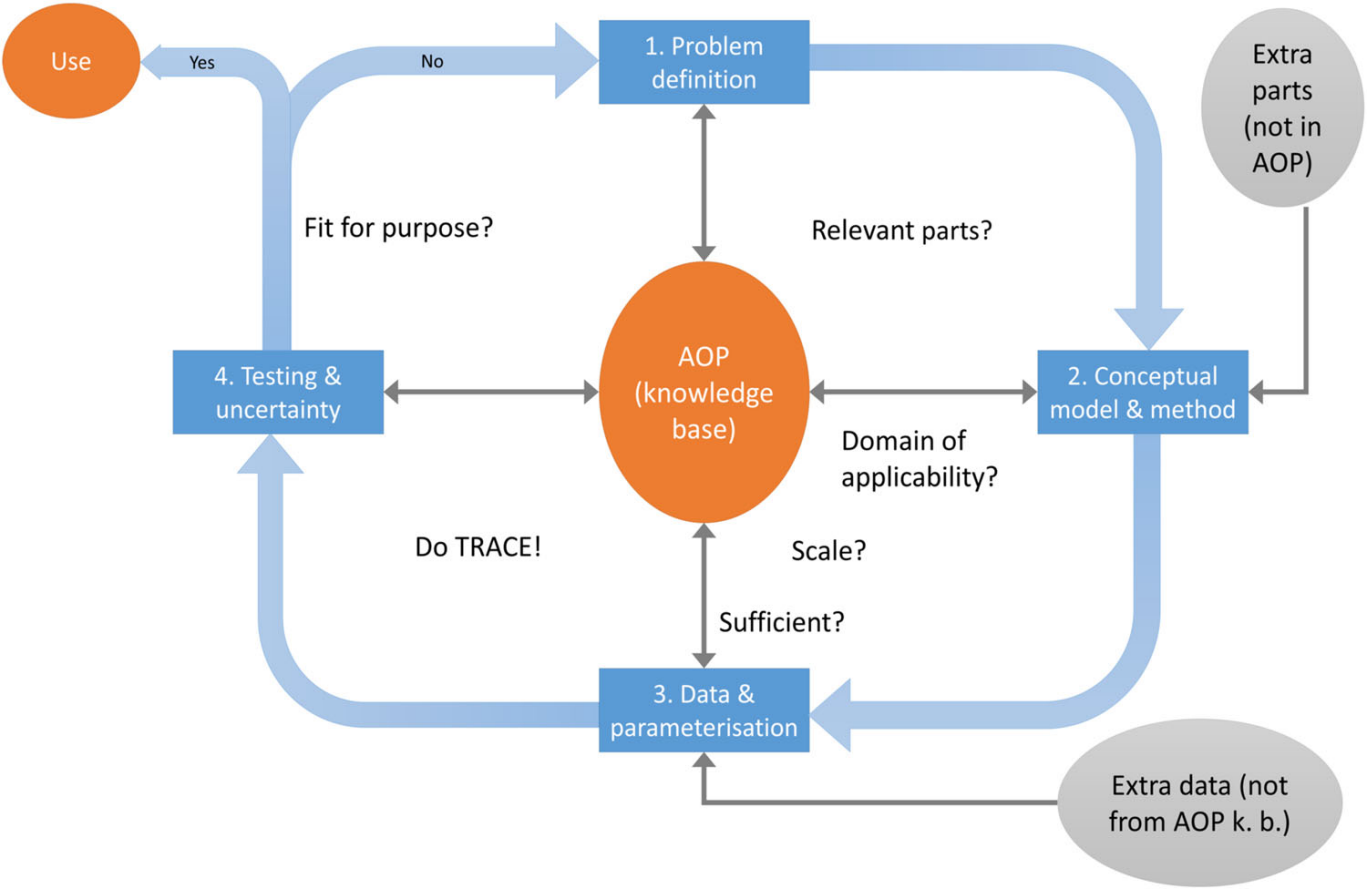
Building a quantitative model based on an adverse outcome pathway (AOP). The modeling cycle (modified from Schmolke et al. 2010) illustrates how the AOP knowledge base (AOP k.b.) can feed into the model development process. The final fit for purpose assessment of the model can be facilitated by the transparent and comprehensive ecological model documentation (TRACE) framework (Schmolke et al. 2010; European Food Safety Authority 2014).

The structure, degree of detail, and confidence needed in a qAOP model greatly depends on the specific question needing to be addressed and what is relevant for decision making (Wittwehr et al. 2016). Although there is no generic qAOP model that is independent from its final use, models representing specific KERs can be developed and used in multiple qAOP models. For example, Conolly et al. (2017) used an oocyte growth dynamics model developed by Watanabe et al. (2016) to model aromatase inhibition leading to reduced fecundity in fish. Because the oocyte model is a stand‐alone component that models egg production as a function of plasma vitellogenin levels in fathead minnows, it could be used in other fathead minnow qAOP models involving vitellogenin and oocyte production. The approach can be applied to other species but would require species‐specific models. Detailed computational qAOP models may require a large dataset for their development, such as the underlying data used for development of the qAOP model for aromatase inhibition (Conolly et al. 2017). Hence, the first steps in developing a qAOP model are formulating the question to be answered, estimating the level of biological fidelity needed, and evaluating whether there is sufficient information available to start the modeling cycle (Figure 2). The applicability domain (i.e., species, life stages, appropriate temporal scale, and biological level of organization) of the underlying AOP must also be examined to ensure that it meets the question requirements.

To formulate the question means that one identifies exactly what should be modeled to support the needs of the end user or decision maker. This will have a strong impact on the type of model that is used. Broadly, qAOPs are likely to be used for 2 categories of questions: 1) to understand and assess the risk of new, untested chemicals to a given species; and 2) to understand and assess the risk of a given (group of) chemical(s) to new, untested species. The questions can include more specific context as to what regulatory action or decision will be made or what protection goals are relevant. For example, if the question is a screening or prioritization issue such as what chemicals have the potential to cause liver toxicity, the qAOP models developed could be simple, could require fewer data, and could have a higher uncertainty because screening/prioritization approaches are not final assessments, However, if the question is “would exposure to a chemical lead to significant risk of liver toxicity?” and the outcome may result in banning of that chemical, then a highly accurate model would need to be developed. Thus, constructing a qAOP that is fit for purpose requires one to start with a clearly defined question/problem with well‐described requirements.

A draft conceptual qAOP model (i.e., the key events and their linkages within an AOP) may be built de novo based on scientific evidence or may be obtained from a qualitative AOP in the AOP knowledge base (Organisation for Economic Co‐operation and Development 2019). The AOP knowledge base is a crowd‐based resource that catalogs AOPs and aggregates the underlying mechanistic information, including supporting weight of evidence for adjacent or nonadjacent KERs. A complete qAOP model describes the links between key events mathematically, to relate the dose–response activation of a molecular initiating event to the response–response dynamics of KERs and the manifestation of an adverse outcome. A qAOP model may not necessarily have the same structure as the AOP it is based on. For example, a qAOP model may explicitly describe response–response relationships of adjacent KERs present in an AOP rather than all KERs, which may help prevent model overfitting and reduce the amount of data needed for model parameterization and testing. Consistent with good modeling practices, one must explicitly describe the quantitative assumptions one is making for the KERs for which quantitative data are lacking. Conversely, and if required, additional events such as feedback loops may be included (Shoemaker et al. 2010; Breen et al. 2013) or complexity added to the description or modeling of a KER. An example for the latter is provided by KERs leading from key events at the level of an individual organism to adverse outcomes at the population level, which can consider biotic (interspecies and intraspecies interactions) and abiotic effects (e.g., temperature), feedbacks, and compensatory processes (Murphy et al. 2008; Forbes and Calow 2012). It is also possible to construct quantitative models for networks of AOPs that share one or more key events and/or KERs (Knapen et al. 2018). Although adding more biological complexity to qAOPs may result in greater biological fidelity, developers of qAOP models should keep in mind that a founding principle of building models is to keep them as simple as possible.

Building qAOP models is not significantly different from building other computational models for decision support, and therefore it is sensible to draw on the experience already available. Good modeling practices that provide detailed guidance on every step of the modeling cycle have been developed for ecological modeling (Schmolke et al. 2010) and for mechanistic effect models that may support risk assessment of plant protection products in the European Union (European Food Safety Authority 2014). The modeling cycle (Figure 2) includes the following steps: problem definition; assembly of a conceptual model and translation into computer code using a modeling method (see the Supplemental Data for different modeling methods); assembly of data and model parameterization; model testing including sensitivity and uncertainty analysis; and, lastly, use of the model for support of decision making. The AOP knowledge base may already provide much of the information used at different steps of the qAOP modeling cycle. Modules inside the AOP knowledge base may be used as a collaborative platform in which mechanistic information, conceptual models, response–response relationships, and supporting quantitative information can be stored and shared (Edwards et al. 2016). Other data may be available from the scientific literature and specialized databases, such as the US Environmental Protection Agency (USEPA) Aggregated Computational Toxicology Online Resource (ACToR), which aggregates data from thousands of public sources on more than 500 000 chemicals (US Environmental Protection Agency 2019). Databases focused on biological modeling (e.g., Chelliah et al. 2013 or The Systems Biology Institute 2019) may also be useful in qAOP model development.

### Data needed to make a qAOP model

The availability of suitable data is arguably the most important requirement in the development of a qAOP model. Quantitative response–response data among the molecular initiating event, key events and the adverse outcome are needed to explicitly model and parameterize each KER. Models intended for use in regulatory decision making should be scientifically sound, robust, and thoroughly tested, and should make valid predictions (European Food Safety Authority 2014). Meeting those expectations requires a level of accuracy that can only be guaranteed by using experimental data of sufficient quality and quantity to support the level of certainty required. The ideal characteristics of the data will vary greatly depending on the type of model being developed, the question being asked, and how accurate the prediction needs to be. For example, the development of simple Bayesian network models may only require enough data to show that inactivation of a particular key event results in the inactivation of a subsequent key event. However, development of models describing multiple, precise KERs would require detailed concentration–response and response–response relationships. Many aspects of the characteristics of data needed to develop models are described in European Food Safety Authority (2014) and in Conolly et al. (2017).

To obtain adequate dose–response data, care needs to be taken to appropriately consider dose ranges and time points. Ideally, this requires a dose range bracketing a dose that elicits no observable response and a dose that elicits a maximal response. In practice, the number of doses will be limited by the available resources but should be a minimum of 5 to establish a dose/response–response relationship. If the focus of the model is on changes over time, then statistical/modeling approaches for time series analysis often require a minimum of 10 time points for successful analysis. However, fewer time points can be sufficient, too, if the data allow the temporal aspects of a given model to be parameterized. Usually the simpler the model the fewer parameters it has and the fewer data points are needed. Ultimately the design of experiments for data collection will depend on the question being asked. For example, models used for prioritizing chemicals for more in‐depth toxicological analysis may not need as much biological fidelity as a model used for determining acceptable levels of chemicals in drinking water. Depending on the complexity of the model, data requirements can be higher than the quantitative data already available from a qualitative AOP description. For certain applications of qAOP models, such as those related to nonlaboratory or endangered species, a paucity of experimental data can be anticipated. In such cases, theoretical relationships or extrapolations from related KERs in other species may be necessary to quantify KERs within the qAOP model specific to the species of interest. In many cases, generation of additional data may be required during the modeling cycle to improve the qAOP model.

The most efficient means to develop a qAOP depends on the question being asked, the data available, and what is known about the relationships between the key event and the adverse outcome. Although every KER within an AOP can be quantified and mathematically described, this may be unnecessary if a molecular initiating event or an early key event has a well‐described statistical relationship to the adverse outcome. For example, highly predictive models have been made for the AOP for membrane disruption (narcosis) leading to respiratory failure whereby a measure of the molecular initiating event (log octanol/water partition coefficient [*K*_OW_]) is significantly correlated with the adverse outcome narcosis (Mackay et al. 2009). Baldwin et al. (2009) used the significant relationship between the molecular initiating event of acetylcholinesterase inhibition and feeding behavior in salmon to create a predictive model to assess the effects of pesticide exposure on the productivity of wild salmon populations.

### Documentation of qAOP model development

Transparent documentation of model development, testing, and analysis is key to increasing user confidence in the model and acceptability for decision making (Schmolke et al. 2010; European Food Safety Authority 2014; Grimm et al. 2014). The European Food Safety Authority scientific opinion on modeling closely follows the TRAnsparent and Comprehensive model Evaluation (TRACE) framework (Table 1), which was originally developed for the use of ecological models in chemical safety assessment (Schmolke et al. 2010), but later broadened to apply to all mechanistic effect models used for ecological risk assessment of chemicals (Grimm et al. 2009; Augusiak et al. 2014; Grimm et al. 2014). The TRACE framework applies to both ecological and human health qAOP modeling applications as well, and its application would ensure that important aspects of model testing and analysis are appropriately described and documented, including model verification, sensitivity analysis, validation, and uncertainty analysis. These are all important aspects to consider when establishing the confidence that can be placed in a model. The modeling cycle closes with an assessment of whether the model is fit for purpose, that is, whether it can be confidently used to answer the question it was designed for.

**Table 1:** Transparent and comprehensive ecological modeling documentation (TRACE) adopted for quantitative adverse outcome pathway (AOP) modeling

Level	Step	Description
Development	Problem formation	Predict an endpoint of regulatory relevance in chemical hazard and risk assessment; estimate which combination of molecular initiating events/key events is required to trigger an adverse effect.
	Model design and formulation (≠ programming)	Decide whether physiologically based pharmacokinetic, toxicokinetic, statistical, or dynamical system models may best describe the quantitative relations required in the anticipated decision‐making context.
	Implementation	Implement the model. A combination of different models targeting the need to describe different key event relationships by different approaches may be considered.
	Parametrization and calibration	Obtain parameters for the different AOP levels from literature or the AOP knowledge base, or by conducting additional experiments. Thresholds that trigger key events or instantiation of differential equations describing relationships represent examples of parametrization.
Analysis	Verification and sensitivity analysis	Test whether the quantitative model adequately describes the relation of molecular initiating event, key event, and adverse outcome and identify parameters that would have the strongest impact on the adverse outcome prediction.
	Validation	Validate the model using different chemicals or other independent data.
Application	Quantification of uncertainties	Compare with experimental data and estimate the deviation, identify data gaps, and propagate parametric and structural uncertainty to predictions.
	Results	Decide whether the confidence is sufficient, and the problem can be addressed.
Repeat	Rerun the steps to optimize the model or adopt the problem formulation (increase feasibility)	Revise and repeat the modeling chain if performance deviates from the expected results.

## GENERAL MODELING APPROACHES FOR qAOP MODEL DEVELOPMENT

The aspect that marks the transition from a descriptive AOP to a qAOP is the degree to which the biology or dynamics underlying response–response relationships are described statistically and/or by a mathematical function in the KERs. The qAOP models can use increasing specification for KERs, ranging from scalar weights to functional relationships (including probabilistic relationships), to entire models specifying how adjacent key events interact. Different modeling approaches can be used depending on data availability and how well the mechanisms underlying the KERs are known (Table 2; detailed descriptions of the modeling approaches are given in the Supplemental Data).

**Table 2:** Modeling approaches for quantitative adverse outcome pathways (qAOPs)^a^

	Description of key event relationship	Relevant models and analyses	Typical data needs	Relevant case studies and applications
AOP	Directed: $KE_{A}\to {\mathrm{KE}}_{B}$ (e.g., all key event relationships in AOP represent a causal linkage)	Causal theory, network/graph analyses techniques	Graph structure providing the connectivity between key events	Steatosis AOP network (Burgoon et al. 2017); graph exploration of AOP networks (Villeneuve et al. 2018)
	Directed and signed relationship: $KE_{A}\overset{\pm }{\to }KE_{B}$ (e.g., increasing and decreasing, i.e.,$↑KE_{A}\Rightarrow ↓KE_{B}$)		Experimental data on supporting key event relationship	Frequently present in AOP knowledge base to support the key event relationship
	Direction and scalar‐weighted relationship: $KE_{A}\overset{\pm w_{A,B}}{\to }KE_{B}$	Weight of evidence models, multicriteria decision analysis, Bayesian analysis	Expert judged weights	Semiquantitative weight of evidence analysis (Becker et al. 2015; Collier et al. 2016)
qAOP	Directional and functional relationship:$KE_{A}\overset{f}{\to }KE_{B}$			
	Probabilistic (e.g., probability of key event activation)	Bayesian networks	Expert or empirically determined probabilities; experimental data on key events	Predicting mode of action (Carriger et al. 2016); predicting states of key event (active, inactive; Burgoon et al. 2017)
	Linear or nonlinear (e.g., saturable response)	Regression modeling	Experimental data on 2 or more key events under different levels of perturbation	Prediction of adverse outcome relationship between a key event (plasma vitellogenin levels) and a downstream key event (fecundity; Miller et al. 2007). Relation amongacetylcholinesterase inhibition, food intake, and growth (Baldwin et al. 2009)
	Time‐resolved	ODE, IBM, LPM	Independent parameter measurement; temporal response data	Predicting temporal response on hypothalamus–pituitary–gonadal axis (Conolly et al. 2017) Dynamic energy budget modeling (Jager et al. 2014)

^a^ For detailed model descriptions, see the Supplemental Data.

KE = key event; ODE = ordinary differential equations; IBM = individual base models; LPM = Leslie projection matrix.

As has been demonstrated by modeling the AOP aromatase inhibition leading to population decline in fathead minnow, it is not necessary to apply a single type of model to build a qAOP model (Conolly et al. 2017). Different KERs and available data often require combining different models to describe the response–response relationships and possibly also the time‐course of the adverse outcome as a function of the degree of activation of the molecular initiating event and the intervening KERs.

## APPLICATION OF qAOP MODELS IN DECISION MAKING

Quantitative AOPs have the potential to support decision making in the development of new chemicals or drugs, identifying and predicting potential chemical hazards, dose–response relations, and risk assessment. Modeling of biological pathways using qAOP models enables the prediction of outcomes based on early events of both single chemicals and mixtures, the inference of exposure levels required to produce an adverse effect, and an understanding of species‐specific differences. We discuss how qAOP models can be used to predict effects of chemicals using relative potencies, how reverse toxicokinetics (rTK) can be combined with qAOP models to estimate hazardous external exposure levels, and different approaches for extrapolation of a qAOP across species. Finally, we present a detailed example of how one can use Bayesian network modeling to develop a qAOP network and how this can be applied to understanding the degree of potential health hazards of individual chemicals and their mixtures. Specific examples of qAOP modeling are currently limited in number due to the relatively recent development of the AOP framework. However, elements similar to those described in qAOP modeling (e.g., molecular initiating events, key events, quantitative KERs, adverse outcomes) can be found in several case studies. We describe these studies as well as hypothetical scenarios in which qAOPs might be applied.

### Extrapolating qAOP models to different chemicals using relative potencies

Computational models that accurately simulate or predict effects of perturbing biological pathways or adverse effects are often established using specific chemicals with known toxicological effects. Although a model can claim to be chemically agnostic, it often uses chemical‐specific parameters such as concentration–molecular initiating event response relationships that permit one to relate the concentration increase in a specific chemical to a predicted outcome. Such a model can be extrapolated to predict effects of other chemicals that also interact with the same molecular initiating event by relating the concentration–response relationship of a new chemical to that of the reference chemical. Conolly et al. (2017) used the relative potency of a new chemical to a reference chemical to extrapolate a qAOP model developed to predict population impacts of one chemical, fadrozole, to predict the response for another, in this case the fungicide iprodione. The qAOP modeled aromatase inhibition (the molecular initiating event) leading to reproductive dysfunction in fathead minnow using 3 computational models: a hypothalamus–pituitary–gonadal axis model (based on ordinary differential equations) of aromatase inhibition leading to decreased vitellogenin production (Cheng et al. 2016), a stochastic model of oocyte growth dynamics relating vitellogenin levels to clutch size and spawning intervals (Watanabe et al. 2016), and a population model driven by fecundity (Miller et al. 2007). The qAOP was modeled on data generated with the potent aromatase inhibitor fadrozole as a stressor and then used to predict potential population‐level impacts. The model was employed to predict iprodione effects on populations using in vitro data for inhibition of aromatase activity from ToxCast (Richard et al. 2016). This was achieved by deriving a toxicity equivalency factor for iprodione relative to fadrozole. Once the relative potency of iprodione to fadrozole was established, the impact of iprodione on fathead minnow population trajectories was then estimated using a read‐across approach with the response–response relationships in the qAOP. This approach is generally applicable when in vitro assays measuring molecular initiating events are available to compare the potency of a new chemical with a model chemical whose performance is known. This has the added advantage of making the use of the qAOP for other chemicals more accessible to users that do not have sufficient expertise to directly model kinetics of new chemicals with the same molecular initiating event.

As with any chemically agnostic qAOP, the aromatase inhibition model developed by Conolly et al. (2017) does not account for differences in the toxicokinetics of a test chemical (e.g., whether the compound metabolized to forms with greater or lesser activity) or differences in bioavailability between in vitro and in vivo assays. This can be refined, if necessary, by coupling a physiologically based pharmacokinetic or toxicokinetic model to the qAOP to add functions describing the compound‐specific effects of adsorption, distribution, metabolism, and excretion on concentrations at the molecular initiating event (Figure 1).

### Combining qAOP models with toxicokinetic models to estimate hazardous external exposure doses

Because qAOPs model dose–response and response–response relationships, they can be used in determining whether a given exposure might result in the occurrence of hazardous effect. For risk assessments, chemical‐specific exposure models are used that describe how much chemical an organism is exposed to as a result of chemical release into the environment (e.g., aggregate exposure pathways; Teeguarden et al. 2016). Linking external chemical exposure levels to a qAOP requires a translation of environmental exposure levels into a relevant internal dose at the site of the molecular initiating event using toxicokinetic models (including physiologically based toxicokinetic models). Unlike qAOPs, toxicokinetic models are chemical specific because they must account for the physical, chemical, and biological interactions of a particular chemical.

**Figure 3:**
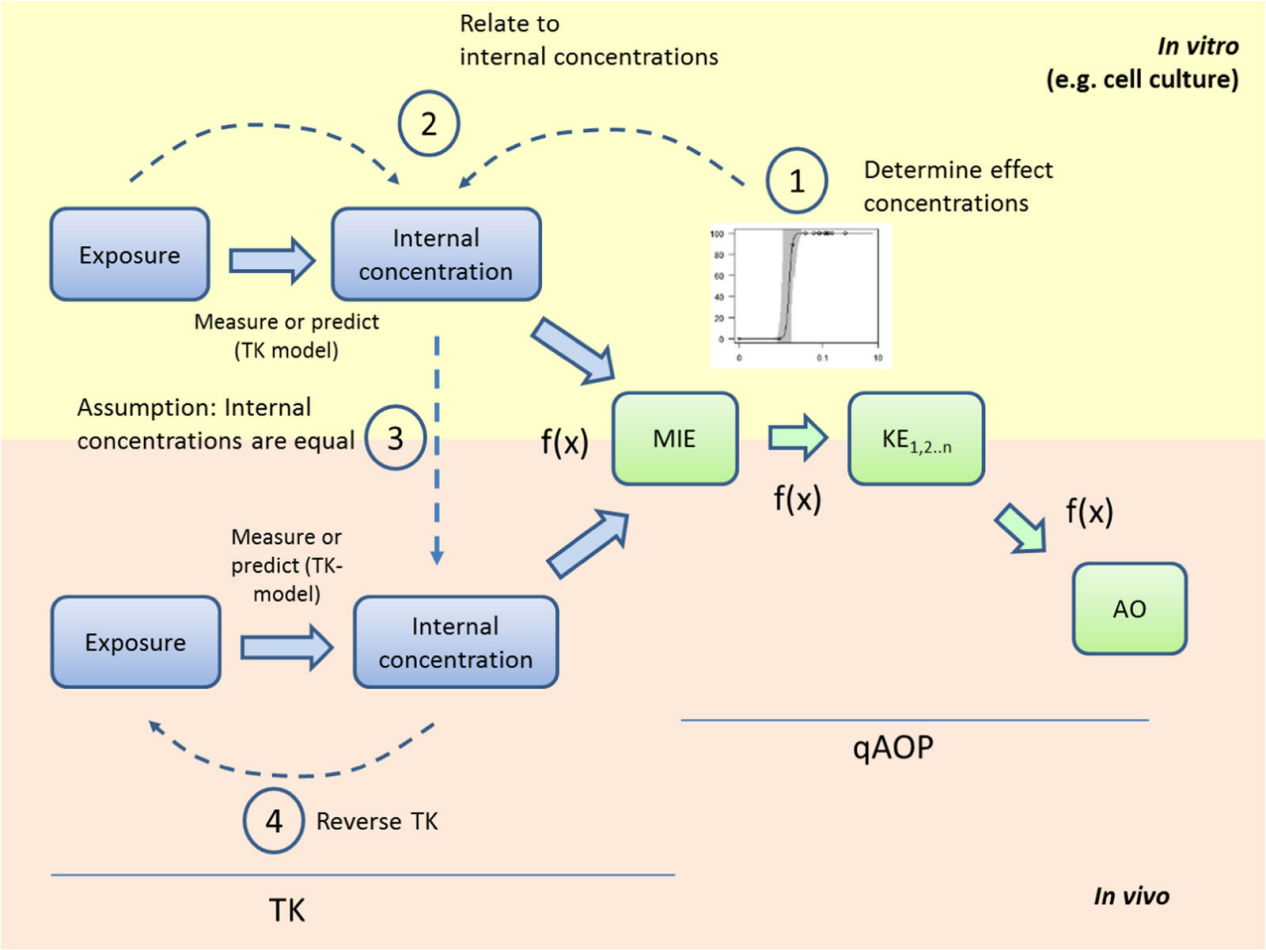
Inference of an external in vivo dosing from an in vitro effect concentration using reverse toxicokinetics (rTK) and quantitative adverse outcome pathways (qAOPs). 1) Concentrations are determined that perturb activities of a molecular initiating event (MIE) or key event (KE) enough to cause significant changes in the final adverse outcome, using modeling or experiments. 2) and 3) The concentrations causing effects in vitro at the molecular initiating event and the adverse outcome are assumed to be the same needed at the in vivo site of action. 4) The predicted in vivo concentration used in combination with reverse toxicokinetics describing metabolism, binding, and clearance functions to determine the external dose required to achieve the internal dose at the molecular initiating event. The boxes represent the different steps involved in toxicokinetics (blue) and AOPs (green). Green arrows represent KERs. Blue arrows represent the time‐sequential links between exposure toxicokinetics. Dashed lines represent different elements of modeling external exposure levels, internal doses, and reverse toxicokinetic modeling. *f*(*x*) = quantitative descriptors.

A major goal in risk management is determining safe levels of chemical exposure. A dose–response assessment can be performed using qAOP models to determine internal concentrations that perturb an molecular initiating event and cause an adverse outcome to occur. Once the concentration causing the point of departure from normal is identified, it can be extrapolated to an in vivo concentration using rTK models (Judson et al. 2011; Wetmore et al. 2015). In vitro to in vivo extrapolation is based on the assumption that an AOP is triggered by the internal bioavailable concentration present at the target site of the molecular initiating event (Figure 3). For example, Stadnicka‐Michalak et al. (2015) used cultured fish cell lines to measure the key event for reduction of cell proliferation, and an rTK model was used to extrapolate levels causing effects in vitro to the corresponding in vivo exposure concentration needed for inhibition of fish growth. Interestingly, the model used by Stadnicka‐Michalak et al. (2015) corresponds to a very compact AOP: the molecular initiating event (reduced cell proliferation) is directly linked to the adverse outcome (reduced fish growth) via a quantitative model.

In vitro to in vivo extrapolation using rTK models has also been used to determine exposure levels needed to cause human skin sensitization (MacKay et al. 2013). The use of rTK modeling enabled the determination of the topical application concentrations that could activate the molecular initiating event of covalent protein modification and, subsequently, predicted to cause the adverse outcome of allergen‐driven skin inflammation. Depending on the route of exposure in the body, rTK models often include functions describing hepatic clearance and plasma binding because distributions by blood and metabolism in the liver often play large roles in the distribution, metabolism, and excretion of chemicals in the body, which in turn dictate chemical concentrations near a molecular initiating event. For example, Judson et al. (2011) and Wetmore et al. (2015) used a high‐throughput approach for rTK modeling that included a one‐compartment model and data from hepatic clearance and plasma protein binding assays. The model was used to predict the human oral dose equivalents that would be needed to cause an effect in vivo—based on the chemical levels found to cause an effect on in vitro assays representing biological pathways. These one‐compartment models may exhibit considerable uncertainty, and more complex compartment models for rTK modeling may provide greater accuracy in extrapolating from in vitro to in vivo effects (Rowland et al. 2017). Nevertheless, the examples show that rTK models can be connected to chemical effect thresholds based on in vitro data or qAOP predictions to estimate external doses needed to cause effects at the adverse outcome.

### Extrapolation of qAOP models across species

Species extrapolation is essential for environmental hazard and risk assessment because data or toxicological assays are not always available for the species of concern. Differences among species (similar to extrapolation from acute to chronic toxicity) are generally accounted for using large uncertainty factors of 10‐ to 1000‐fold to ensure safe exposure thresholds (Ashauer and Jager 2018). The qAOP models could be used to refine our understanding of species differences by basing cross‐species extrapolations on mechanistic species‐specific information. A qAOP model incorporating species differences may be useful in specifying the size of the uncertainty factor for extrapolation between species. The predictions of the extrapolated qAOP can be validated by comparison with the results of exposing the species of interest to chemical stressors under laboratory conditions. Understanding these differences can also be used to determine whether a standard risk assessment approach with large uncertainties is appropriate.

Given a conserved AOP structure, the qAOP for one species could be adapted to another by modifying species‐specific dose–response or response–response parameters such as binding affinity and activation of a receptor. This is similar to the idea of adjusting mechanistic effect model parameters based on species traits (Rubach et al. 2011). For example, the AOP for aromatase inhibition (Conolly et al. 2017) could be extrapolated from fathead minnow to other fish species by calibrating the model to species‐specific binding affinities, kinetic rates, and hormone concentrations (Murphy et al. 2009; Gillies et al. 2016). Calibration to other species should be relatively straightforward because a limited number of measurements for molecular initiating events or key events and their response–response relationships (here chemical binding affinity to aromatase, kinetics of aromatase inhibition, and resulting plasma hormone levels) would be needed rather than response–response measurements for every KER. Furthermore, species‐specific toxicokinetic differences can impact on the level of internal bioavailable concentrations and the external effect concentrations required to elicit an adverse outcome. In the case of aromatase inhibition, predictions of hormone production from the extrapolated qAOP can be compared with measured plasma hormone concentrations to assess the reliability of the extrapolated qAOP.

Predictive qAOP models for cross‐species extrapolations could also be created when there is ambiguous mechanistic information available for KERs by using a simple statistical model relating the change in the molecular initiating event to the adverse outcome. Substituting a statistical correlation with a mechanistic function may increase the uncertainty of the final prediction because a mechanistic approach also captures biology features such as the feedback control underlying the adverse response. Uncertainty can also be created by the development of more complex models that describe more KERs in a qAOP. However, the uncertainty generated by a complex model reflects the variability of the underlying biology that determines the outcome of a model.

Conservation of protein sequences among species can also be used to infer how species differ in affinity or how sensitivity in a species of interest varies from model species (Gunnarsson et al. 2008). The aryl hydrocarbon receptor (AhR) presents an example in which differences in the amino acid of the target among avian and fish species result in different binding affinities to dioxin‐like compounds and, as a result, different dose–response relationships at the molecular initiating event (Farmahin et al. 2014; Doering et al. 2015). A qAOP model for activation of AhR leading to embryonic mortality could be extrapolated across multiple species including fish, birds, and other taxa by modifying the molecular initiating event (AhR activation) dose–response function in the model to account for species sensitivity to dioxin‐like compounds (Doering et al. 2013). The analysis of similarity for specific genes and proteins across a few or many species is facilitated by tools such as sequence alignment to predict across species susceptibility (SeqAPass) that look at available sequences from as many species as possible (LaLone et al. 2016). Provided that species‐specific information on molecular initiating events and KERs is available for selected species, a sensitivity distribution and the chemical of interest using a qAOP model could be made.

It is difficult to determine whether slight differences in sequence homology may result in increased or decreased binding affinity and whether those differences are biologically significant. Information on the functional homology of proteins from molecular docking simulations may be more accurate in predicting binding affinity differences when only slight changes are found in protein sequences (Ballester and Mitchell 2010). Sequence similarities can also be used to identify conserved key events in a pathway, providing support that data or modes from one species could be used for another.

Incorporation of toxicokinetic information can be crucial for application of qAOP models in cross‐species extrapolation. Allometric scaling is a simple approach to account for differences in body size and how such differences change concentrations at the target site (Espié et al. 2009). Allometric scaling could be improved by combining it with in vitro data prediction of toxicokinetics (Lavé et al. 1999). However, such scaling may not account for certain differences in the activity and specificity of biotransformation enzymes, protein binding, and other toxicokinetic properties that can cause chemical concentrations to differ considerably among species. As a result, more detailed pharmacokinetic modeling is needed to capture differences in metabolic transformation capacity (Espié et al. 2009). For example, pharmacokinetic modeling was used to extrapolate to humans a rat biologically based computational model for nasal cell carcinoma in formaldehyde‐exposed rats (Conolly et al. 2003). Conolly et al. (2004) adapted the rat model to humans by replacing the model component describing rat nasal airways with components describing the entire respiratory tract in the human model. The resulting inhalation model was then used to predict regional formaldehyde dosimetry throughout the respiratory tract and to link the dosimetry to time‐course effect data on cell division (as a surrogate for cell death); the model was finally used to predict human respiratory tract tumor responses to inhaled formaldehyde. Using this type of approach, models from one species can be extrapolated to another by substituting model components such as the ones describing distribution of formaldehyde in the respiratory system.

Differences in toxicokinetics between different species and/or different life stages can affect whether data from one species can be used to predict effects in another species. A prominent example is the application of zebrafish as a screening tool for human toxicology (Bambino and Chu 2017) or the use of mammalian data for wildlife species toxicology (Huggett et al. 2004). Clearly, differences in exposure routes would need to be considered when a qAOP model‐based prediction is applied, for example, to account for differences in concentrations at the target site. For instance, qAOP modeling has been used to show that the therapeutic levels of glucocorticoids in humans can be linked to various effects observed in exposed fathead minnow, by incorporation of pharmacokinetic and pharmacodynamic differences (Margiotta‐Casaluci et al. 2016).

Fish embryos are attractive models for predicting mammalian adverse effects because of their small size and amenability to high‐throughput testing. The use of fish embryos adds a further level of complexity because the pharmacokinetics and key parameters of KERs may differ not only between species but also between life stages. A particular area of high relevance is the prediction of human/mammalian developmental toxicity using chemical impacts on fish embryo development (Sipes et al. 2011; Brannen et al. 2016).

When an AOP structure in a species of interest is unknown at levels below the cellular or organism level but has known similarities with a model species at a high‐level key event such as behavior, whole‐organism models could be used to extrapolate qAOP models. This can be accomplished by coupling models describing chemical impacts on individual organism growth, reproduction, or behavior, such as dynamic energy budget (DEB) models (Jager et al. 2006; Jager et al. 2014; Baas et al. 2018) to models that extrapolate effects of contaminants or stressors on behavior to population endpoints such as individual based models (Murphy et al. 2008), or matrix models (Diamond et al. 2013). Modeling approaches such as DEB are applied at the whole‐organism level to predict how flow of energy from food is diverted to different functions including reproduction, growth, and repair. Suborganismal processes and ecotoxicogenomic measurements such as gene expression, vitellogenin levels, or lipid levels can be used as key events and also to determine energy distribution in DEB models and, in the process, connect key events to population‐level endpoints (Ananthasubramaniam et al. 2015; Murphy et al. 2018b). When qAOP outputs are linked to DEB model input parameters, then these changes can be compared with known values of these parameters for a given or multiple species and used to extrapolate population effects. Models using DEBs have been developed for a wide range of animals including rare species (such as the right whale) to predict the accumulation of lipophilic contaminants and effects on growth and reproduction (Klanjscek et al. 2007; Murphy et al. 2018b), and explore how life history affects these processes in marine mammals as a whole (Noonburg et al. 2010). Coupling of qAOP models to DEB models for rare species may enable extrapolation of in vitro testing results to understand the potential impacts of chemicals on rare and endangered species like marine mammals. However, identifying the physiological mode(s) of action may still pose a challenge and should be a topic of future research to support hazard or risk assessment (Ashauer and Jager 2018; Murphy et al. 2018a).

### Modeling AOP networks for hazard screening of chemicals and chemical mixtures

Prioritization and screening applications are designed to identify chemicals that are likely to cause an adverse outcome at a given exposure concentration so that they can be subjected to further, more in‐depth, testing. Prioritization and screening efforts often use in vitro assays to assess the potential of chemicals to cause an effect such as endocrine disruption, neurotoxicity, or skin sensitization. Bayesian networks provide one approach by which experimental measurements (e.g., in vitro screening assays, omics, or biochemical or toxicological data) can be aligned with AOPs to determine the probability that a chemical can activate an adverse outcome (for more information on Bayesian networks and their use, see Neapolitan 2004).

**Figure 4:**
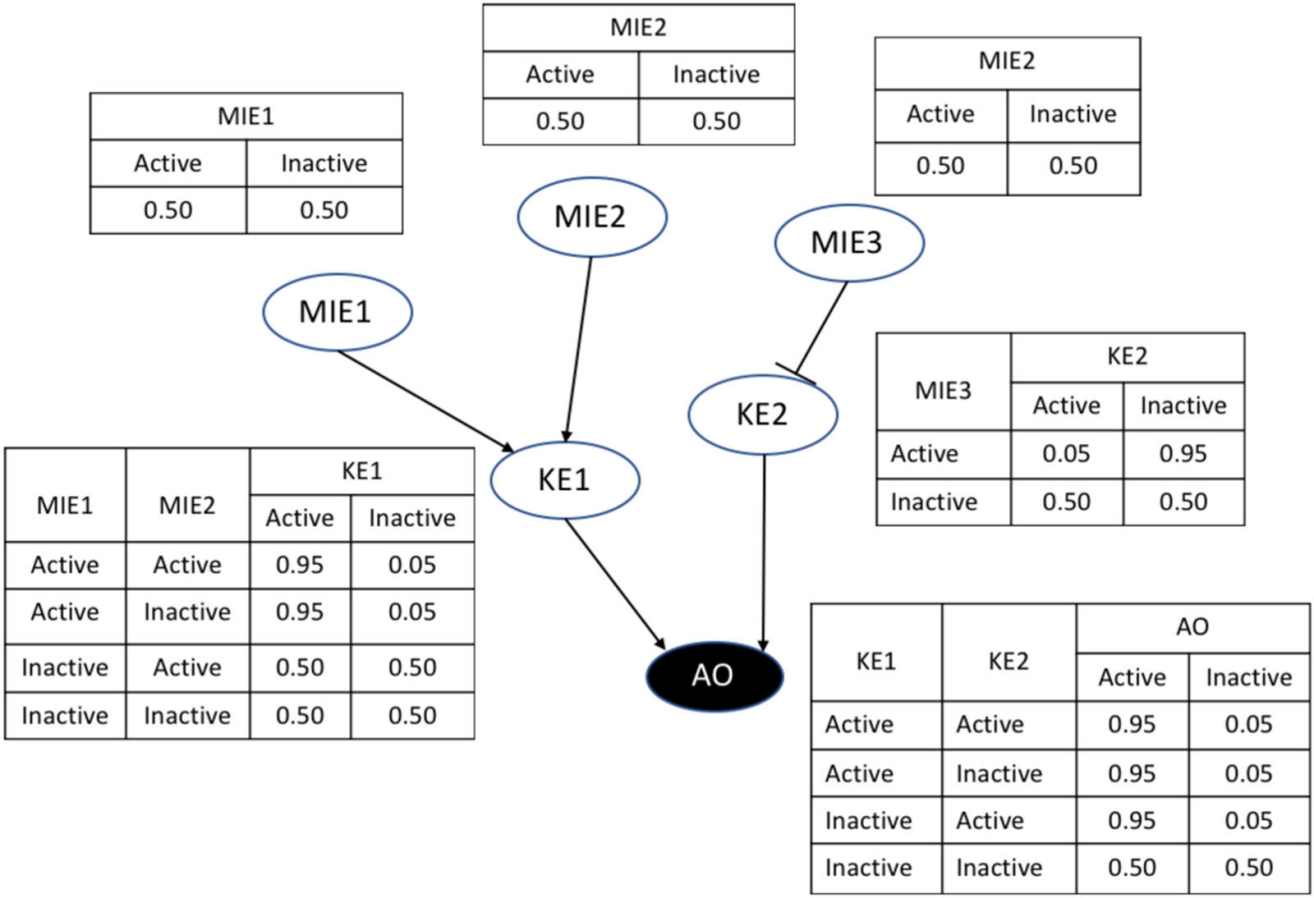
Scheme of a hypothetical binary adverse outcome pathway (AOP) Bayesian network. Tables associated with nodes describe the probability that a node (molecular initiating event [MIE], key event [KE], or adverse outcome [AO]) is active or inactive given the state of the upstream nodes. The final output of the model is the probability that an adverse outcome is active or inactive.

Bayesian network analysis is a relatively simple approach toward modeling complex situations that could be particularly useful when quantitative information is limited and/or when the adverse outcome of concern could be caused by multiple pathways. This is true especially when other modeling approaches are not feasible. Like graphical representations of AOPs, a quantitative AOP Bayesian network (qAOPBN) model is a causal network in which molecular initiating events, key events, and adverse outcomes are represented by nodes whose activity can be measured, for example by an in vitro assay, and whose edges represent causal relationships between nodes (Figure 4). The probability that an upstream node(s) will activate or inactivate a downstream node is defined experimentally or by expert judgment informed by available data and literature and summarized in probability tables associated with each node (Figure 4). Depending on the available information, a qAOPBN can have a high level of uncertainty, which may be acceptable depending on the application and question to be answered.

A qAOPBN uses information on the activation of each node across the network to model the potential for a chemical to cause the adverse outcome. For example, Jaworska et al. (2013) and Pirone et al. (2014) used a Bayesian network approach to screen chemicals for the potential to cause skin sensitization by integrating event measurements from in vitro assays and computational models to estimate the potency of a chemical to cause skin sensitization. The different assays were related to the AOP for skin sensitization (Organisation for Economic Co‐operation and Development 2012). For each assay, thresholds were defined such as a 150% induction of a cell surface marker or a 50% viability reduction in keratinocytes. In silico data such as the presence of a certain structural alert or predicted activity were considered as well. The data were finally used to derive an integrated testing strategy with a Bayesian network, and probabilities were determined by analysis of a dataset of 124 chemicals.

**Figure 5:**
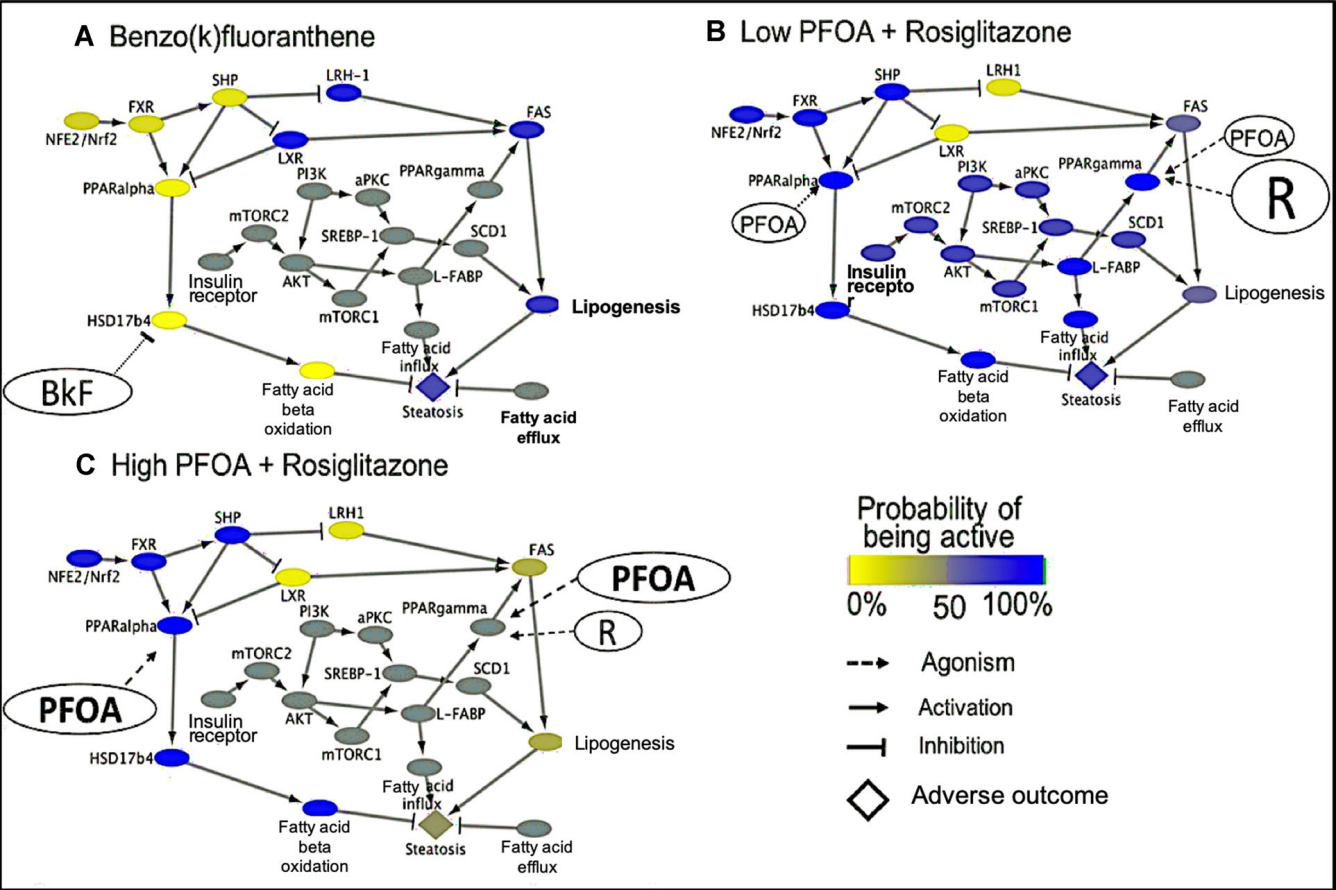
Example of quantitative modeling of chemical impacts on liver steatosis adverse outcome pathway (AOP) networks using a Bayesian network approach. (**A**) Effect of benzo[*k*]fluoranthene (BkF) inhibition of HSD17b4 on the liver steatosis AOP network. (**B**) Interaction of perfluorooctanoic acid (PFOA) at concentrations found in the environment with rosiglitazone (R) at therapeutic levels on the liver steatosis AOP network. (**C**) Mixture interactions on the liver steatosis AOP network when PFOA is at high concentrations relative to rosiglitazone‐contaminated water. Ovals represent chemicals, molecular initiating events, and key events. Arrows represent causal relationships through which an upstream event activates a downstream event. T bars represent causal relationships through which an upstream event inhibits a downstream event. The diamond node represents the adverse outcome of steatosis. Yellow nodes equal a 0% probability of being active, gray nodes equal a 50% probability of being active, and a blue nodes equal a 100% probability of being active. aPKC = atypical protein kinase C; AKT = serine/threonine‐protein kinase; FXR = farnesoid X receptor; L‐FABP = liver‐type fatty acid binding protein; LRH‐1 = liver receptor homolog 1; LXR = liver X receptor; mTORC = mammalian target of rapamycin complex; NFE2/Nrf2 = nuclear factor erythroid 2/NFE2‐related factor 2; PI3K = phosphoinositide 3‐kinase; PPAR = peroxisome proliferator‐activated receptor; SCD1 = stearoyl CoA desaturase 1; SHP = small heterodimer partner; SREBP = sterol regulatory element binding protein.

The skin sensitization Bayesian network included variables that did not represent key events in the skin sensitization AOP, and it represents an application of Bayesian networks to a linear AOP (branched, but with one molecular initiating event and one adverse outcome; Organisation for Economic Co‐operation and Development 2012) rather than a network of AOPs. Bayesian networks can be particularly useful for more complex situations, that is, networks of AOPs (Burgoon et al. 2017; Knapen et al. 2018). One example of an adverse outcome involving a network of AOPs is the well‐studied adverse outcome of liver steatosis. In steatosis, fatty acids accumulate in the liver, resulting in nonalcoholic fatty liver disease that can lead to cirrhosis of the liver (Tuyama and Chang 2012). Steatosis can be caused by changing the activity of 4 critical key events (fatty acid efflux, fatty acid uptake, lipogenesis, and peroxisomal fatty acid β‐oxidation; Angrish et al. 2016; Burgoon et al. 2017). In a companion paper (Burgoon et al., US Army Corps of Engineers, unpublished manuscript), we have constructed a steatosis qAOPBN (Figure 5). The steatosis AOPBN was established as a binary Bayesian network where only 2 states, active or inactive, are used as input (based on the measured state of an event) for the molecular initiating event or key event and as output for the adverse outcome. Probability tables were constructed using expert judgment and available evidence in the literature to determine whether a node was active or inactive based on the state of the adjacent upstream nodes. When no information was available, the probability of activation was set at 50%. When evidence strongly supported the possibility that perturbing a molecular initiating event or key event would change the adjacent key event or adverse outcome, probabilities were set close to 100% to account for the degree of uncertainty that occurs in biological measurements. The Bayesian network algorithm then uses the probability tables for each node to determine the probability of activity for parent and child nodes using Bayes’ rule.

To illustrate how AOP networks and Bayesian network modeling could be used to assess the hazards of single chemicals and chemical mixtures, the steatosis qAOPBN was perturbed with different chemicals, and the potential to cause steatosis was assessed (Figure 5). For example, concentrations of benzo[*k*]fluoranthene greater than 0.5 µM have been shown to inhibit or inactivate activity of peroxisomal β‐oxidation of fatty acids by the enzyme HSD17b4, a key event in the steatosis qAOPBN (Burgoon et al. 2017). Inhibition of the key event for HSD17b4 results in a probability of 1% that the key event for fatty acid β‐oxidation will be activated. The resulting accumulation of fatty acids is associated with a high probability that steatosis will be activated (Figure 5A). The probability that steatosis will be activated can then be compared with thresholds of the probability of activation set by decision makers or risk managers to decide whether benzo[*k*]fluoranthene should be investigated further to confirm its ability to cause steatosis.

Understanding the potential of chemical mixtures to cause an adverse outcome is a complex effort. The AOPBNs may be particularly useful in understanding the potential hazards of chemical mixtures because, in an AOP network, multiple molecular initiating events and pathways are described that can be affected by multiple chemicals acting on common or different molecular initiating events. As an illustration, using the steatosis example, consider the impact of rosiglitazone, an antidiabetic drug that is a full agonist of peroxisome proliferator‐activated receptor γ (PPAR‐γ), which can activate steatosis (Lehmann et al. 1995), and perfluorooctanoic acid (PFOA), a highly stable chemical with widespread human exposure and uptake (Fry and Power 2017), which is a partial agonist of PPAR‐γ and a full agonist of PPAR‐α, which inhibits steatosis by increasing the β‐oxidation of fatty acids (Vanden Heuvel et al. 2006). These chemicals have the potential to interact when diabetic patients being treated with rosiglitazone drink water that is contaminated with PFOA (Figure 5B). In the presence of therapeutic levels of rosiglitazone and environmental concentrations of PFOA, PPAR‐γ is expected to be active because rosiglitazone will outcompete PFOA to occupy PPAR‐γ binding sites due to the higher efficacy of rosiglitazone in activating PPAR‐γ (Vanden Heuvel et al. 2006; Fry and Power 2017). Activation of PPAR‐γ ultimately results in an increase in the probability of steatosis activation, despite activation of PPAR‐α by PFOA, which would normally inhibit steatosis. This result is consistent with observations of increased steatosis in clinical studies of rosiglitazone in obese patients (Massart 2017) and manifestations of steatosis in mice fed a high‐fat diet in combination with rosiglitazone (Gao 2016).

A critical aspect in chemical mixture interactions is how different ratios of chemicals can cause different effects. For example, when healthy people are exposed to both PFOA and rosiglitazone through contaminated water, PFOA is likely to be at much higher concentrations than rosiglitazone. We assume that external exposure concentrations reflect internal concentrations at the molecular initiating events and that toxicokinetics do not interfere. This would result in PFOA outcompeting rosiglitazone for occupancy of the PPAR‐γ receptor, increasing PPAR‐α activation and decreasing the probability of steatosis occurring (Figure 5C). These predictions are consistent with experimental studies in which reduction of PPAR‐γ activity in obese mice, via antagonism or gene knockout, combined with activated PPAR‐α, resulted in decreased steatosis (Morán‐Salvador et al. 2011; Zhang et al. 2014; Shiomi et al. 2015).

Our examples demonstrate how qAOP models can be used to assess impacts of both individual chemicals and mixtures. A significant source of uncertainty in applying an AOPBN is the accuracy of expert judgment in determining probabilities and thresholds when they cannot be inferred from datasets. Nevertheless, the ability to rapidly develop and integrate data from different sources into the results of an AOPBN has many practical applications and is useful in developing qAOP networks for screening and prioritization of chemicals. The uncertainty surrounding probabilities can be reduced by using more complex relationships or datasets, but the relatively simple assumptions and binary input/outputs we have applied in our examples can be used for screening and prioritization.

## CONCLUSIONS AND FUTURE PERSPECTIVES

Quantitative AOP models can provide a bridge from descriptive knowledge to the prediction of an adverse outcome in hazard and risk assessments. Quantitative approaches cover a spectrum of methods, taking AOPs from purely descriptive to highly defined quantitative models. At present, examples of qAOP models are scarce but show considerable promise for real‐world applications. Given this high potential for chemical regulation, it can be expected that quantitative approaches will continue to be developed. However, it is crucial that qAOP models have properly defined application domains, documentation, and testing support to avoid misuse and poor regulatory acceptance. The ability of models to predict outcomes and answer regulatory questions must be well tested and documented using good modeling practices so that one can clearly understand how reliable a model's predictions are or what type of improvements are required to make them more reliable. Purpose‐specific validation may include validating the ability of a model to predict whether a chemical has a potential to cause an adverse outcome. This requires the availability of adverse outcome data to anchor in vitro assay responses of traditional toxicity endpoints to adverse effects of regulatory concern. For example, the USEPA Endocrine Disruptor Screening Program validated an estrogen receptor agonist model for screening by examining its ability to accurately predict estrogen receptor agonists from a library of well‐characterized chemicals (Judson et al. 2017). In the future we may lack data for traditional experimental regulatory toxicity. In this case, one may consider that these data could be generated for a selected set of compounds to validate a new qAOP model and endpoint of regulatory concern.

In practice, a number of potential limitations of qAOP models need to be considered: 1) pathways other than the AOP modeled may be biologically more significant in causing the outcome; 2) species and life‐stage differences might be outside the applicability domain of the AOP; and 3) limitations in the complexity of the population models could interfere with translation of individual effects to population outcomes (lack of density dependence, other life stages, ecological factors, etc.). Given that the concentration at the target site will be critical to estimate the degree of an adverse effect with qAOP models, uncertainties and differences in the toxicokinetics in vitro and in vivo are important parameters that could impact the prediction of hazards given external exposure concentrations. Approaches such as using data from in vitro assays to incorporate effects of mixtures on specific events or qAOP networks for integrating multiple pathways and multiple chemicals are promising for addressing mixture effects. However, great challenges remain in predicting and understanding mixture effects under realistic environmental scenarios because the amount and quality of relevant, mechanistic data are even more demanding than for a single chemical. There are lessons from related fields that can aid qAOP model development and regulatory adoption, for example, the TRACE documentation. The most important issue is a clear definition of the question to be answered with the qAOP model, and this must be articulated before undertaking model development. To support transparency, understanding, and acceptance, models need to have clear and detailed documentation of data use and sources, model development and coding, rigorous testing (e.g., comparison of a qAOP model prediction with independent data), and communication of the assumptions, applicability, and limitations of the model.

## Supplemental Data

The Supplemental Data are available on the Wiley Online Library at DOI: 10.1002/etc.4505.

## Disclaimer

The views and opinions expressed in the present review are those of the authors and do not necessarily reflect the official policy or position of the associated organizations or their employers.

## Data Accessibility

The article is a review and hence is mainly based on the available literature. Occasional reference is made to recent partially unpublished data (e.g., Figure 5), which will be provided on request to the corresponding author (stefan.scholz@ufz.de).

## Supporting information

This article includes online‐only Supplemental Data.

Supporting information.
