# Supplementary material for: Building and Applying Quantitative Adverse Outcome Pathway Models for Chemical Hazard and Risk Assessment
Source: Environ Toxicol Chem. 2019 Aug 8;38(9):1850–65. doi: 10.1002/etc.4505 (PMC6771761; doi:10.1002/etc.4505)
Supplement: Supplementary file 1 — Supporting information. [file ETC-38-1850-s001.pdf]

Supplement to the article

Building and Applying Quantitative Adverse Outcome Pathway Models for Chemical Hazard and Risk Assessment

Index

S1. Mathematical models that can be used to develop qAOPs 1
S1.1. Bayesian Network Models 1
S1.2. Regression Models 2
S1.3. Ordinary Differential Equation models 3
S1.4. Agent or Individual Based Models 3
S1.5. Dynamic Energy Budget Models 4
S1.6. Population models 5
S2. References 5

S1. Mathematical models that can be used to develop qAOPs

S1.1. Bayesian Network Models

A Bayesian Network modeling approach enables development of large network models that incorporate multiple, interacting AOPs to predict a common AO. In Bayesian qAOPs, connected KEs are assumed to be conditionally dependent, while the KEs without direct connection are independent, which simplifies the calculation of joint probability distribution over the events (MIE, KE, AO) of the network. This establishes response-response relationships that can vary from simple binary states (e.g. active vs non-active) to multiple categories, such as levels of potency. For example, Jaworska et al (Jaworska et al., 2013) applied a Bayesian Network modeling approach to predict dose-response relationships of chemicals tested in several in vitro assays representing different KEs within the AOP for skin

sensitization by using the categories non-sensitizer, weak, moderate or strong and extreme.

Bayesian networks can be used to predict the likelihood that a KE or AO occurs based on the state of (other) KEs within the network, to classify chemicals based on responses of in vitro assays representing KEs, and even *de novo* inference of an AOP network structure based on experimental data and network learning algorithms (Carriger et al., 2016; Jaworska et al., 2013). To create Bayesian network models within a qAOP modelling context, an acyclic AOP network and a set of conditional probability tables for each KE in the network are required. Within a AOP network, a cycle is a path between KEs that traverses the same KE more than once. When a network is acyclic, there are no cycles present. For further discussion of directed acyclic graphs, which many AOP networks can be categorized as, see Bang-Jensen and Jorgen (2008) . Conditional probability tables describe the probability that a downstream KE occurs given the degree of perturbation of an upstream KE. Probabilities relating the response relationships between upstream and downstream KEs can be derived from experimental data indicating causality (e.g. knock down of KE expression or pharmacological blocking of activity) or response-response dependence (e.g. both upstream and downstream KE activities are measured in the same experiment over a concentration range or over a time series). Bayesian network approaches are flexible in that simple models with binary states (e.g. KEs as active/inactive or above/below a threshold) can be developed rapidly and more complex models with greater fidelity developed if sufficient resources are available.

S1.2. Regression Models

If data describing the responses of two or more KEs measured in the same experiment is available, one can also develop regression models. Regression models are a large class of statistical models used to estimate the relationships between measured variables, (e.g. the outcome that one wants to predict and predictors (measured data that used to predict the outcome)). Values for parameters in the model are generally estimated by fitting the model to a subset of experimental data, while a different subset is used for validation. .

In an AOP context, the predictor variables could be measurements of particular KEs used to predict a downstream KE or an AO. Data for regression models should include measurements of at least two different KEs collected from the same experiment over a range of response-response relationships. For example, Miller et al. (2007) developed a regression model of fathead minnow plasma VTG and fecundity to predict population sustainability based on VTG levels in plasma. Regression models may be used alone, precede or be established secondary to other models such as ordinary differential equations (discussed in the following section), since they are easier to understand and do not require more sophisticated computational/modeling skills (Conolly et al., 2017).

S1.3. Ordinary Differential Equation models

Ordinary Differential Equation models (ODEs) can incorporate temporal effects and systems dynamics. For ODE-based qAOPs it is therefore necessary to understand how the elements of an AOP evolve over time). ODE modeling is particularly well suited to incorporating complex system dynamics, such as signaling cascades, feedback and feedforward loops and, in particular, the effect of time-dependent perturbations (e.g. acute vs chronic). ODE models can be applied practically at any level of biological organization, from biomolecular interactions (e.g. steroidogenesis and signal transduction, Shoemaker et al., 2010; Cheng et al., 2016)), to cellular dynamics (e.g. T-cell proliferation, Baker et al., 1997) to population dynamics (e.g. ecological modeling, Hallam et al., 1983)

ODE-based models require detailed mechanistic knowledge but in many cases it may not be necessary (from a predictive standpoint) or even desirable (from a data needs or model coding and performance standpoint) to include all KEs and KERs, but rather to focus on the parts for which data availability enable ODE modelling and where it is required to answer the question of interest.

S1.4. Agent or Individual Based Models

Individual- or Agent-based models (ABMs) track the behaviors of individual agents over time and the aggregated properties of the agents describe key demographic metrics for populations, such as survival, production, and movement (Grimm and Railsback, 2005). Agents can represent a wide range of items including molecules, cells in tissues, and individuals within a population. They are particularly

useful when the individual differences between agents can lead to different responses to the same stimulus, for instance, when two animals of the same species have a different reaction to exposure to the same chemical. ABMs are also used when interactions among individuals are crucial to understanding the system, and when it is necessary to include adaptive behavior that could include physiology and/or energy budgets (DeAngelis and Grimm, 2014). In the AOP context, ABMs have been shown to be particularly useful for modeling AOs related to individual behavior and the translation of individual behavioral responses to adverse effects at the population level. For example, Murphy et al. included MeHg induced swimming behavior and reactive distance alterations, both key events measured on individual larval fish, into an ABM calibrated for Atlantic croaker and determined the effects of these behavioral alterations on cohort survival and growth, which are population relevant endpoints (Murphy et al., 2008). Because ABMs are models of individuals, they require substantial data at the level of individuals to make them more informative than models that assume all individuals of a similar grouping (eg., age) are identical (Caswell, 2001).

S1.5. Dynamic Energy Budget Models

Dynamic energy budget (DEB) models consider an organism as a system that assimilates energy and mass into an animal and describes how these are used internally to maintain or execute various physiological functions. DEB models are based on ODEs describing time varying measures of growth, reproduction, maintenance and other physiological function (Kooijman, 2010; Jager et al., 2014) - and the potential impact by environmental stressors. These ODE models are attractive because they are based on over 30 years of metabolic theory and are composed of relatively few parameters that can be used to describe a broad spectrum of species with diverse life history patterns. The DEBtox approach has been used to link external chemical concentrations to life history traits (Jager and Zimmer, 2012),and to examine the toxicological effects of chemicals on a wide range of animals (eg effects of nonylphenol in marine polychaetes or uranium on *C. elegans*, (Jager and Selck, 2011; Goussen et al., 2015)).

Recent work has focused on linking the mechanistic pathway based information in qAOPs to the energy partitioning information in DEB models. In this approach, either the qAOP is incorporated into the DEB model by altering the DEB

model itself to accommodate sub-organismal feedbacks, by adding a separate module to DEB to represent a qAOP, or by altering the DEB rates by statistical correlations from KEs (Murphy et al., 2018). Once parameter changes described by qAOPs are mechanistically linked to DEB, DEBs can then be used to examine how toxic chemicals change energetic tradeoffs amongst physiological processes and the impact on life history traits within a whole organism. The cross-talk between qAOP and DEB models would improve the predictive power of qAOP models, and place KEs into a framework that would allow for extrapolation to population level effects by embedding them into ABMs (Martin et al 2013).

S1.6. Population models

The impact on the population represents the ultimate target and regulatory concern for decision making particularly in the field of ecotoxicology. Hence, qAOP models may need to incorporate appropriate population models that estimate the impact of individual performance on the dynamics and structure of a population, via impacts on individual survival and reproduction (Kramer et al., 2011). They may be applied to the final trajectories of diverse AOPs that will converge at the level of the individual, usually in the form of survival, growth or reproductive effects. Population models may accommodate multiple AOs from single or multiple stressors (Diamond et al., 2013), but can also allow us to bridge suborganismal/organismal KEs by e.g. extrapolating the effects of contaminants on behavior to population endpoints (Murphy et al., 2008). Population models take on many forms, such as unstructured ordinary-differential equation models (e.g. logistic population growth; Barnthouse, 2004)), structured demographic models such as matrix projection models (Miller et al., 2007), and individual based models (Railsback et al., 2009). There are many reviews that discuss the use of appropriate population models for different purposes related to risk assessment (e.g. (Galic et al., 2010) and many of these models can be potentially linked to AOPs as well.

S2. References

Baker, C.T.H., Bocharov, G.A., Paul, C.A.H., 1997. Mathematical Modelling of the Interleukin-2 T-Cell System: A Comparative Study of Approaches Based on Ordinary and Delay Differential Equation. Computational and Mathematical Methods in Medicine. 1**,** 117-128.

Bang-Jensen, J., Gutin, G.Z., 2008. 2.1. Acyclic Digraphs. Digraphs: Theory, Algorithms and Applications. Springer, Heidelberg, pp. 32-34.

Barnthouse, L.W., 2004. Quantifying population recovery rates for ecological risk assessment. Environ. Toxicol. Chem. 23, 500-508.

Carriger, J.F., Martin, T.M., Barron, M.G., 2016. A Bayesian network model for predicting aquatic toxicity mode of action using two dimensional theoretical molecular descriptors. Aquat. Toxicol. 180, 11-24.

Caswell, H., 2001. Matrix population models. Wiley Online Library.

Cheng, W.Y., Zhang, Q., Schroeder, A., Villeneuve, D.L., Ankley, G.T., Conolly, R., 2016. Computational Modeling of Plasma Vitellogenin Alterations in Response to Aromatase Inhibition in Fathead Minnows. Toxicol Sci. 154, 78-89.

Conolly, R.B., Ankley, G.T., Cheng, W., Mayo, M.L., Miller, D.H., Perkins, E.J., Villeneuve, D.L., Watanabe, K.H., 2017. Quantitative Adverse Outcome Pathways and Their Application to Predictive Toxicology. Environ. Sci. Technol. 51, 4661-4672.

DeAngelis, D.L., Grimm, V., 2014. Individual-based models in ecology after four decades. F1000prime reports. 6.

Diamond, S.L., Murphy, C.A., Rose, K.A., 2013. Simulating the effects of global climate change on Atlantic croaker population dynamics in the mid-Atlantic Region. Ecol. Model. 264, 98-114.

Galic, N., Hommen, U., Baveco, J.M., van den Brink, P.J., 2010. Potential application of population models in the European ecological risk assessment of chemicals II: Review of models and their potential to address environmental protection aims. Integr. Environ. Assess. Manag. 6, 338-360.

Goussen, B., Beaudouin, R., Dutilleul, M., Buisset-Goussen, A., Bonzom, J.-M., Péry, A.R.R., 2015. Energy-based modelling to assess effects of chemicals on Caenorhabditis elegans: A case study on uranium. Chemosphere. 120, 507-514.

Grimm, V., Railsback, S.F., 2005. Individual-based modeling and ecology. BioOne.

Hallam, T.G., Clark, C.E., Lassiter, R.R., 1983. Effects of toxicants on populations: a qualitative approach I. Equilibrium environmental exposure. Ecol. Model. 18, 291-304.

Jager, T., Barsi, A., Hamda, N.T., Martin, B.T., Zimmer, E.I., Ducrot, V., 2014. Dynamic energy budgets in population ecotoxicology: Applications and outlook. Ecol. Model. 280, 140-147.

Jager, T., Selck, H., 2011. Interpreting toxicity data in a DEB framework: A case study for nonylphenol in the marine polychaete Capitella teleta. J. Sea Res. 66, 456-462.

Jager, T., Zimmer, E.I., 2012. Simplified dynamic energy budget model for analysing ecotoxicity data. Ecol. Model. 225, 74-81.

Jaworska, J., Dancik, Y., Kern, P., Gerberick, F., Natsch, A., 2013. Bayesian integrated testing strategy to assess skin sensitization potency: from theory to practice. J. Appl. Toxicol. 33, 1353-1364.

Kooijman, S.A.L.M., 2010. Summary of concepts of Dynamic Energy Budget theory for metabolic organisation. Cambridge University Press.

Kramer, V.J., Etterson, M.A., Hecker, M., Murphy, C.A., Roesijadi, G., Spade, D.J., Spromberg, J.A., Wang, M., Ankley, G.T., 2011. Adverse Outcome Pathways and ecological risk assessment bridging to population-level effects. Environ. Toxicol. Chem. 30, 64-76.

Miller, D.H., Jensen, K.M., Villeneuve, D.L., Kahl, M.D., Makynen, E.A., Durhan, E.J., Ankley, G.T., 2007. Linkage of biochemical responses to population-level effects: a case study with vitellogenin in the fathead minnow (Pimephales Promelas). Environ. Toxicol. Chem. 26, 521-527.

Murphy, C.A., Nisbet, R.M., Antczak, P., Garcia-Reyero, N., Gergs, A., Lika, K., Mathews, T., Muller, E.B., Nacci, D., Peace, A., Remien, C.H., Schultz, I.R., Watanabe, K., 2018. Linking adverse outcome pathways to dynamic energy budgets: a conceptual model. In Garcia-Reyero, N. and Murphy, C.A., (eds), A Systems Biology Approach for Advancing Adverse Outcome Pathways for Risk Assessment. Springer in press. in: Garcia-Reyero, N., Murphy, C.A., Eds.), A Systems Biology Approach for Advancing Adverse Outcome Pathways for Risk Assessment. Springer, pp. 281-302.

Murphy, C.A., Rose, K.A., del Carmen Alvarez, M., Fuiman, L.A., 2008. Modeling larval fish behavior: scaling the sublethal effects of methylmercury to population-relevant endpoints. Aquat. Toxicol. 86, 470-484.

Railsback, S.F., Harvey, B.C., Jackson, S.K., Lamberson, R.H., 2009. InSTREAM: the individual-based stream trout research and environmental assessment model.

Shoemaker, J.E., Gayen, K., Garcia-Reyero, N., Perkins, E.J., Villeneuve, D.L., Liu, L., Doyle, F.J., 2010. Fathead minnow steroidogenesis: in silico analyses reveals tradeoffs between nominal target efficacy and robustness to cross-talk. BMC systems biology. 4**,** 89.
